# UPLC-TOF-MS/MS-Based Metabolomics Analysis Reveals Species-Specific Metabolite Compositions in Pitchers of *Nepenthes ampullaria*, *Nepenthes rafflesiana*, and Their Hybrid *Nepenthes* × *hookeriana*

**DOI:** 10.3389/fpls.2021.655004

**Published:** 2021-04-21

**Authors:** Muhammad Aqil Fitri Rosli, Ahmed Mediani, Kamalrul Azlan Azizan, Syarul Nataqain Baharum, Hoe-Han Goh

**Affiliations:** Institute of Systems Biology, Universiti Kebangsaan Malaysia, Bangi, Malaysia

**Keywords:** carnivorous plant, flavonoid, hybrid, LC-MS, metabolomics, *Nepenthes*, pitcher

## Abstract

Hybridization is key to the evolution and diversity of plants in nature. Nepenthaceae comprises a family of diverse tropical carnivorous pitcher plant species with extensive hybridization. However, there is no study to date on the metabolite expression of hybrids in this family. We performed a non-targeted metabolomics analysis of the pitchers of two *Nepenthes* species with different dietary habits, namely, the semi-detritivorous *N. ampullaria* and carnivorous *N. rafflesiana* with their hybrid (*N*. × *hookeriana*) for a comparative study. The whole-pitcher samples were extracted in methanol:chloroform:water (3:1:1) via sonication-assisted extraction and analyzed using ultra-performance liquid chromatography time-of-flight mass spectrometry (UPLC-TOF-MS) followed by data analysis to profile chemical compositions. A total of 1,441 metabolite features were profiled from the three species in which 43.3% of features in the hybrid samples were not found in either of its parents. The partial least squares discriminant analysis (PLS-DA) found 324 metabolite features with variable in projection (VIP) values greater than one in which 55 features were statistically significant. This showed that the hybrid is closer to *N*. *rafflesiana*, which is consistent to the previous study on gene and protein expressions. A total of 105 metabolites were putatively identified with manual searches using public metabolite databases. Phenols were detected to be the most abundant secondary metabolites due to a high flavonoid content, especially in *N. rafflesiana*. The most abundant feature 476.3s:449.102 was found to be the most significant VIP for distinguishing between the three species as a chemical marker. This is the first study comparing metabolites in the carnivory organs of different *Nepenthes* species with comprehensive profiling and putative identification. The differential metabolite compositions in the pitchers of different species might have ecological implications with the hybrid showing intermediate phenotype between the parents as well as manifesting unique metabolites. However, there is no clear evidence of metabolites related to the differences in dietary habits between the hybrid and the two parent species.

## Introduction

*Nepenthes* L. is the sole genus of paleotropical carnivorous pitcher plants from the monotypic Nepenthaceae family of Caryophyllales order that is highly distributed around the warm and humid equatorial countries, such as Indonesia, Philippines, and Malaysia (Adam, 1997). *Nepenthes* pitcher plants thrive on poor soils and acquire limiting nutrients mainly from insect prey via the unique fluid-filled pitcher organ developed at the leaf tip connected by a tendril, which allows prey trapping, digestion, and absorption (Clarke and Robinson, 2018). Fascinating pitcher shapes and sizes have been observed in this diverse family of carnivorous plants with over 150 extant species (Murphy et al., 2020), which are linked to the various ecological adaptations related to nutrient acquisition or dietary habits. For example, *Nepenthes ampullaria* Jack have evolved a semi-detritivorous habit with a vestigial pitcher lid bent away from the pitcher opening surrounded by a relatively flat peristome and growing on the forest floor to ease the trapping of fallen leaf litter, thus less dependent on catching prey (Moran et al., 2003; Pavlovič et al., 2011). In comparison, a typical carnivorous *N*. *rafflesiana* Jack produces larger pitchers with attractive coloration and a functional lid covering the pitcher opening, some of which secrete fragrance and sweet nectars to lure insect prey (Di Giusto et al., 2010). Additionally, the viscoelastic fluid (Bazile et al., 2015) and slippery epicuticular wax crystals of the inner pitcher wall with the anisotropic effect of lunate cells (Wang et al., 2018) also contribute to the success of prey capture and retention.

Hybridization is the formation of organisms by cross-fertilization between individuals of different species, which is key to the evolution and diversification of 30–70% flowering plants (Soltis and Soltis, 2009). Hybrids generally express phenotypes intermediate to those of parents, which sometimes result in heterosis or hybrid vigor such that the hybrids survive better than both parents, but the opposite is also possible (Orians, 2000; Goulet et al., 2017). Studies comparing the metabolite expression of hybrids with that of their parent species are limited, but existing evidence suggests that hybrids may express more diverse combinations of secondary metabolites or produce unique phytochemicals novel to both parental species (Cheng et al., 2011; Lätti et al., 2011). This leads to an interesting question on the expression of metabolites in the hybrids of *Nepenthes* species.

Hybridization in the species-rich Nepenthaceae is extensive, either naturally or artificially driven by high horticultural demand as ornamental plants. According to Harry James Veitch in 1906 (Veitch, 1979), the first *Nepenthes* artificial hybridization was initiated by John Dominy who produced *N*. × *dominicana* from *N. rafflesiana* with an unnamed species in 1862, followed by *N*. × *hybrida* from *N*. *khasiana* with an unnamed Bornean species in 1866. Despite the long history, studies on *Nepenthes* hybrids and their chemical compositions are generally limited. Studies of *Nepenthes* have largely focused on the proteins of pitcher fluids (Ravee et al., 2018). Recent studies have reported the effects of endogenous protein depletion on protein secretion in pitcher fluids of *N.* × *ventrata* (Wan Zakaria et al., 2019) and gene expression in pitchers of *N*. *ampullaria* with transcriptome analysis (Goh et al., 2020). The same proteomics informed by the transcriptomics approach was applied to explore the expression of genes and proteins in the pitchers and fluids, respectively, in *N*. *ampullaria*, *N. rafflesiana*, and their hybrid *N.* × *hookeriana* (Zulkapli et al., 2017; Zulkapli et al., 2021).

To date, information is only available on certain targeted metabolites that have been successfully isolated and characterized from some *Nepenthes* species. Advanced metabolite identification using high-performance liquid chromatography (HPLC), nuclear magnetic resonance (NMR), and gas chromatography–mass spectrometry (GC-MS) was performed on a few *Nepenthes* species in several studies, but the isolated and characterized compounds were targeted (Eilenberg et al., 2010; Kováčik et al., 2012). There is only one recent untargeted metabolite profiling study comparing the feeding effect on leaf blades and pitchers of *N.* × *ventrata* based on cheminformatics fingerprinting without compound identification (Dávila-Lara et al., 2020). The authors found little impact of feeding on the metabolite composition of pitchers with only small changes in the polar metabolites.

The current study aims to unveil the effects of hybridization in pitcher plants based on comparative metabolic profiling through an untargeted approach for identifying metabolites. Liquid chromatography–mass spectrometry (LC-MS) is used to classify and compare chemical profiles between *N.* × *hookeriana*, a natural hybrid with its parents, *N. ampullaria* and *N. rafflesiana*. The parentage of *N.* × *hookeriana* was previously discovered based on its morphological resemblance to *N. ampullaria* and *N. rafflesiana* (Danser, 1928), which was confirmed by molecular markers, including amplified fragment length polymorphism (AFLP), random amplified polymorphic DNA (RAPD), and inter-simple sequence repeats (ISSR) (Kiew et al., 2003; Yulita and Mansur, 2012). Differences in the morphology and diet between *N. ampullaria* and *N. rafflesiana* may lead to differences in the metabolite compositions. Therefore, this study provides a comprehensive metabolite profiling of these three species to investigate how the parental molecular phenotypes are manifested in *N.* × *hookeriana*.

## Materials and Methods

### Pitcher Sampling

Pitcher tissues of closely related lowland *Nepenthes* species, *N. ampullaria* Jack and *N. rafflesiana* Jack (Bunawan et al., 2017) with their hybrid *N.* × *hookeriana* Lindl., all of which were originally transplanted from Endau-Rompin National Park, were sampled from an experimental terrace at Universiti Kebangsaan Malaysia (2°55′12.7″N, 101°46′59.7″E) (Supplementary Figure 1). The pitchers were monitored and measured throughout their development (Supplementary Figure 2) to capture the timing of pitcher opening. Pitchers after 7 days of lid opening were chosen as they are reported to be fully functional traps (Bauer et al., 2009). The sampling was performed in the morning between 09:00 and 11:00 h during October to December 2015 with at least four biological replicates from each species (Rosli et al., 2018). Whole pitchers were harvested without the tendril, emptied, and rinsed with deionized water before rapidly frozen in liquid nitrogen and stored at −80°C.

### Phytochemical Extraction

The whole-pitcher samples excluding the tendril were ground until fine homogenous powders using pestle and mortar prechilled with liquid nitrogen, collected in a Falcon tube, and freeze-dried for 48 h. Dried powder samples (100 mg) were extracted using 200 μL of methanol:chloroform:water (3:1:1) via the sonication-assisted method (Shyur et al., 2013; Rosli et al., 2017). Samples were vortexed, sonicated in a water bath sonicator at room temperature for 15 min with maximum frequency, vortexed again, and then centrifuged at 10,000 *g* for 10 min. The extracts were filtered through a 0.22-μm PTFE membrane syringe filter before stored at −80°C.

### Liquid Chromatography-Mass Spectrometry (LC-MS) Analysis

The chromatographic separation was carried out using a Thermo Scientific C18 column (Acclaim^^TM^ Polar Advantage II, 3 × 150 mm, 3 μm particle size) on UltiMate 3000 UHPLC (Dionex) system according to previous reports (Goh et al., 2016; Rosli et al., 2017). The gradient elution was performed for 22 min total run time, using (A) water containing 0.1% formic acid, and (B) 100% acetonitrile as the mobile phase with 0.4 mL/min flow rate at 40°C. The gradient was started at 5% solvent B for 3 min (0–3 min), then increased to 80% solvent B for 7 min (3–10 min) and maintained at 80% solvent B for 5 min (10–15 min). Finally, the gradient was returned to 5% solvent B in 7 min (15–22 min). The injection volume for each sample was 1 μL, and the samples were prepared by dissolving 10 μL of sample extracts in LC-grade methanol (990 μL) spiked with internal standards not present in the studied sample, namely, ribitol, riboflavin, and vanillin at a concentration of 50 ppm each with known basic characteristics, such as retention time (rt) and exact mass. Internal standards are important in metabolic profiling analysis serving as references for relative quantitative analysis and validation on the performance of chromatographic and MS systems (Stobiecki and Kachlicki, 2013). At least four independent biological replicates of pitcher extracts from each species were analyzed.

High-resolution mass spectrometry analysis was carried out using the Bruker Daltonics MicrOTOF-Q III with the following settings: capillary voltage at 4,500 V, nebulizer pressure at 1.2 bar, drying gas flow at 8 L/min with the source temperature at 200°C, and m/z range from 50 to 1,000 Da. The mode of electrospray ionization (ESI) was compared preliminary before the metabolite profiling LC-MS analysis. Most of the metabolites identified through negative mode were present in the total ion chromatogram of positive mode (data not shown). Thus, the positive ESI mode was selected following a higher number of metabolites profiled in preliminary LC-MS analysis and other studies (Goh et al., 2016; Rosli et al., 2017; Dávila-Lara et al., 2020). Independent from the MS analysis of individual samples, the MS/MS analysis was performed for each species using the pooled replicates of all extracts at equal amounts using automated fragmentation settings (Auto-MS/MS) over a mass-to-charge precursor ion ranged between 500 and 1,000 Da (Vargas et al., 2016).

### Mass Spectrometry Data Processing

Mass spectrometry data processing was done according to Krug et al. (2008) with some modifications. The mass spectrometry raw data was extracted from Bruker DataAnalysis (version 4.1) and aligned using Bruker Compass ProfileAnalysis (version 2.1). The advanced bucketing setting was selected with the following parameters: Δm/z = 20 mDa, Δrt = 10 s, threshold of signal-to-noise ratio = 5, and smoothing width = 4. The correlation coefficient was set at 0.7. The exact mass and predicted molecular formula obtained from SmartFormula, a feature in ProfileAnalysis (version 2.1), was selected based on the highest score with low molecular mass error tolerance range (Δppm = 5) and manually matched to accessible public databases, including METLIN^1^ developed for QTOF instruments (Guijas et al., 2018), MassBank^2^ the longest standing community database (Horai et al., 2010), and MetFrag^3^ (Ruttkies et al., 2016) for putative metabolite identification.

Putative metabolites were retrieved from the databases for having molecular weights within the molecular mass error tolerance range of Δppm < 20 to the query m/z values via positive mode adduct. Full-scan LC-MS data were acquired for statistical analysis to shortlist molecular ions with significant differences between samples, followed by independent precursor-ion (PI) scans to acquire MS/MS data of the PIs. These MS/MS data of precursor m/z and retention time were used to derive the structural information with confidence level 1 (CL1), while metabolite features without MS/MS data were putatively identified at confidence level 2 (CL2) according to the Metabolomics Standards Initiative (Blaženović et al., 2018). Erroneous metabolite identifications were removed through manual inspection based on chemotaxonomy information with reference to the KNApSAcK species–metabolite relationship database (Afendi et al., 2012).

### Statistical Analysis

XCMS version 3.7.1^4^ was used for a preliminary inspection of feature detection using the mzXML files and default parameters (ID#6675) optimized for “UPLC/Bruker Q-TOF pos” to generate the statistics of identified features and mirror plots of pairwise comparisons between the samples (Huan et al., 2017). For further statistical analysis, the data matrix generated from ProfileAnalysis (version 2.1) comprising peak intensity of each retention time (rt) and m/z value was normalized to the intensity of the most consistent internal standard (vanillin) using MetaboAnalyst 3.0^5^, a free online tool for statistical and pathway analyses (Xia and Wishart, 2016). The normalized peak intensity data from MetaboAnalyst was exported into SIMCA-P+ (version 14.1, Umetrics, Umeå, Sweden) for multivariate analysis (MVA). The data was preprocessed with Pareto (Par) scaling which was applied to the normalized data to reduce the effects of background noise interference on sample clustering. MVA based on non-supervised principal component analysis (PCA) and supervised partial least squares discriminant analysis (PLS-DA) were applied to cluster samples according to the profiled metabolite features and to identify distinct metabolites that differentiate between the three species. One-way analysis of variance (ANOVA) with a significance of *P* < 0.05 was conducted on the normalized peak intensity data to differentiate the statistically significant metabolites among the three species. The variable importance in the projection (VIP) ranks the overall contribution of each variable to the PLS-DA model, and those variables with VIP > 1.0 were considered relevant for group discrimination (Xie et al., 2008). The list of important VIPs was based on PLS-DA loading plots with VIP scores > 1. Venn diagrams were generated using a web tool^6^.

## Results

### Morphological Changes During Pitcher Development

**FIGURE 1:**
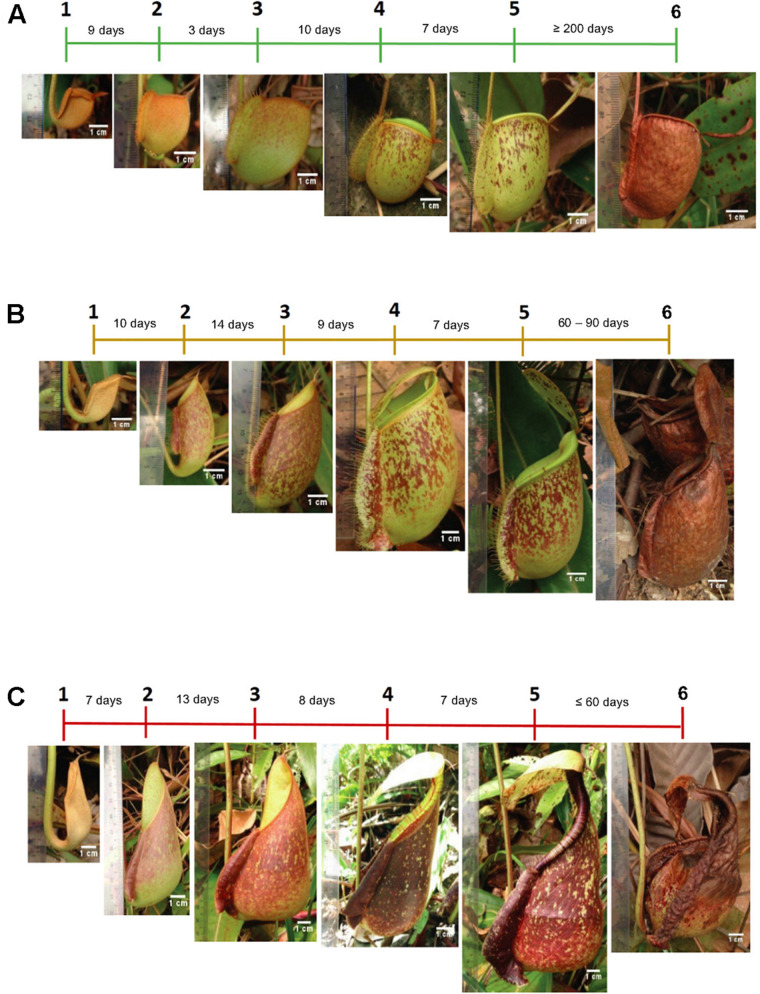
The timeline of pitcher morphological changes of **(A)** *N. ampullaria*, **(B)** *N.* × *hookeriana*, and **(C)** *N. rafflesiana*. Different phases and durations of pitcher development are indicated in the timeline.

For the sampling of pitchers, daily observations of morphological changes were performed *in situ* (Supplementary Figure 1) throughout pitcher development (Figure 1). The three species showed different pitcher longevity with *N*. *ampullaria* pitchers being the most long-lived (>6 months) compared to the short-lived (≤2 months) *N*. *rafflesiana* pitchers with the hybrid pitchers showing intermediate longevity (2–3 months). It took around 1 month (22–33 days) from pitcher initiation at the tendril tip to pitcher opening for all three species. Pitcher development continues after opening and maturity within 7 days with a stable morphology, namely, the positioning of the lid, peristome structure, coloration, and pitcher size (Figure 1 and Supplementary Figure 2). Therefore, 7-day-old mature pitchers after lid opening were chosen to standardize sampling for metabolomics analysis.

### Metabolite Profiling

**FIGURE 2:**
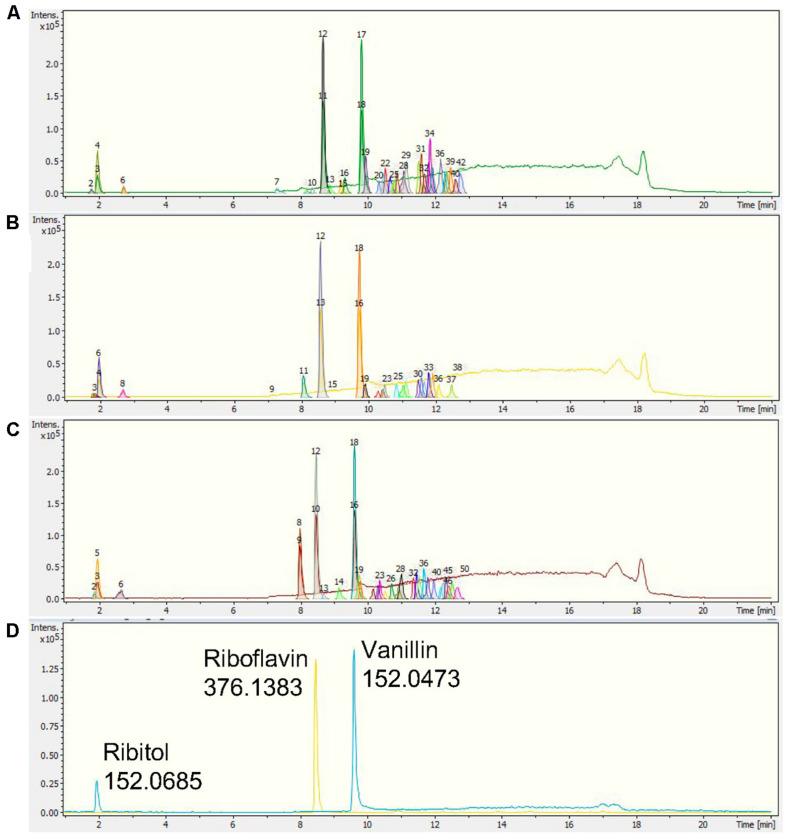
Representative base peak chromatograms (BPCs) with extracted ion chromatograms (EICs) of **(A)** *N. ampullaria*, **(B)** *N.* × *hookeriana*, **(C)** *N. rafflesiana*, and **(D)** a blank with internal standards, namely, ribitol (1.9 min), riboflavin (8.4 min), and vanillin (9.5 min).

The chromatographic separation of the three *Nepenthes* samples and internal standards can be visualized on a base peak chromatogram (BPC), which shows the peak intensity based on the m/z value and retention time (rt) with extracted ion chromatogram (EIC). All three internal standards were successfully identified in the chromatogram, including ribitol at rt 1.9 min, riboflavin at rt 8.4 min, and vanillin at rt 9.5 min (Figure 2). Based on a comparison of all the chromatograms, all three internal standards were present and separated at similar rt in each sample. Samples from the three species showed metabolic separation patterns with different numbers of metabolite features. There were metabolite features uniquely present in one species or all species with different intensities. For instance, a metabolite feature with the highest intensity after internal standards at rt 8 min exhibited the highest intensity in *N. rafflesiana*, moderate in the hybrid, *N*. × *hookeriana*, and the lowest in *N. ampullaria*.

A statistical analysis was performed using XCMS using the raw data from all the samples for pairwise comparisons (Supplementary Figure 3) and feature detection (Supplementary Figure 4). Comparison between the two parent species showed the biggest difference with 22 significant differentially accumulated features (DAFs), followed by the hybrid and *N*. *ampullaria* (9 DAFs), and lastly between the hybrid and *N*. *rafflesiana* (2 DAFs) (Supplementary Figure 3).

**FIGURE 3:**
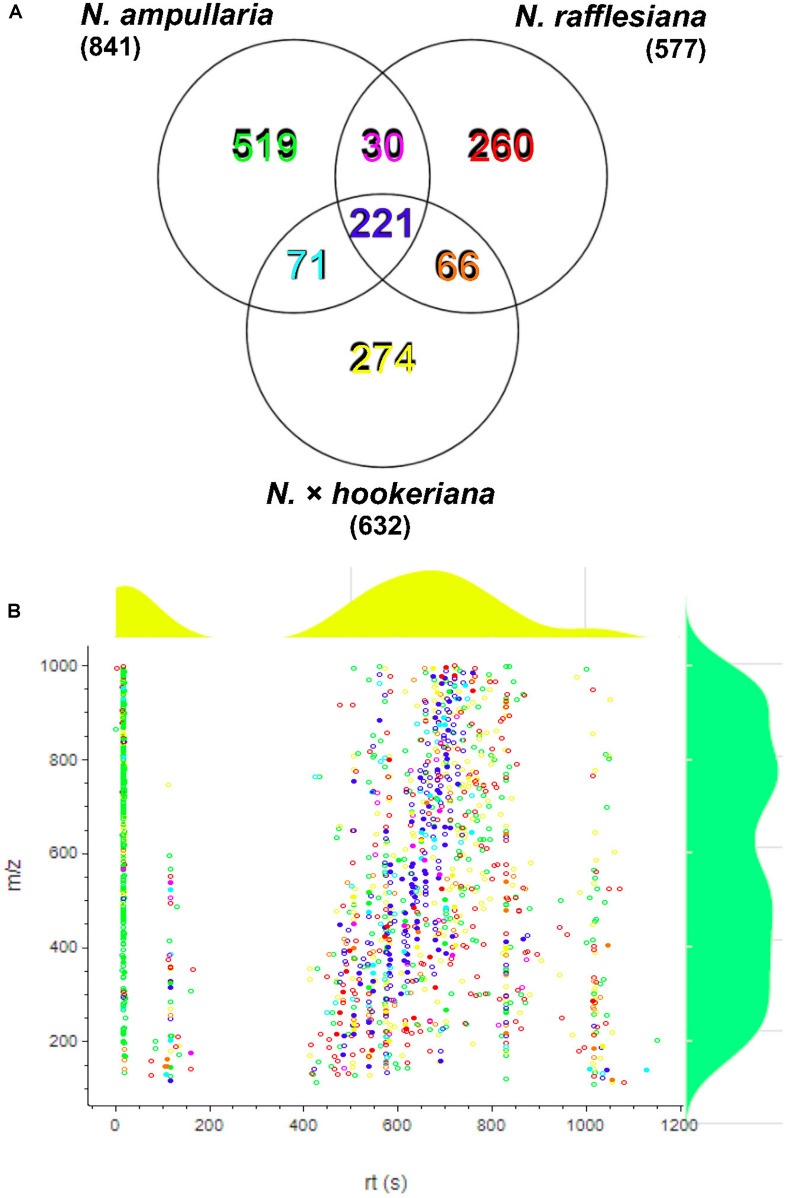
Metabolite profiling. **(A)** Venn analysis of metabolite features identified from LCMS analysis of all three species. Numbers in parentheses show the total number for each species. **(B)** Scatter plot of all metabolite features with overlaying relative density plots for retention time (rt) along the X-axis and mass over charge (m/z) along the Y-axis. Symbol colors correspond to the font colors in the Venn diagram. Filled symbols represent features with putative identification.

After filtering and bucketing using Bruker Compass ProfileAnalysis, we successfully profiled a total of 1,441 metabolite features in pitcher samples from all three species (Supplementary File 1). The distribution of pitcher metabolite features for each species is shown in a Venn diagram (Figure 3A). The pitcher extracts with the highest number of profiled metabolite features were found in *N. ampullaria* with 841 features, followed by the hybrid species, *N*. × *hookeriana* (632) and *N. rafflesiana* (577). Only 221 metabolite features were present in all three species with many features unique to individual species. It is noteworthy that there were 274 (43.3%) features found in *N.* × *hookeriana* which were not found in any of the parent species. Similar numbers of metabolite features were shared by the hybrid with *N. ampullaria* (292, 46.2%) and *N. rafflesiana* (287, 45.4%). However, there were many more unique metabolite features in *N. ampullaria* (549, 65.3%) that were absent in the hybrid, compared to *N. rafflesiana* which shared 49.7% of its total metabolite features with the hybrid. The mass-to-charge ratio (m/z) of metabolite features were rather evenly distributed compared to the retention time (rt) (Figure 3B). Most of the unique features found in *N. ampullaria* were detected during early rt (25 s), while the majority of the shared features were found in the middle of rt (400–800 s).

### Multivariate Analysis

**FIGURE 4:**
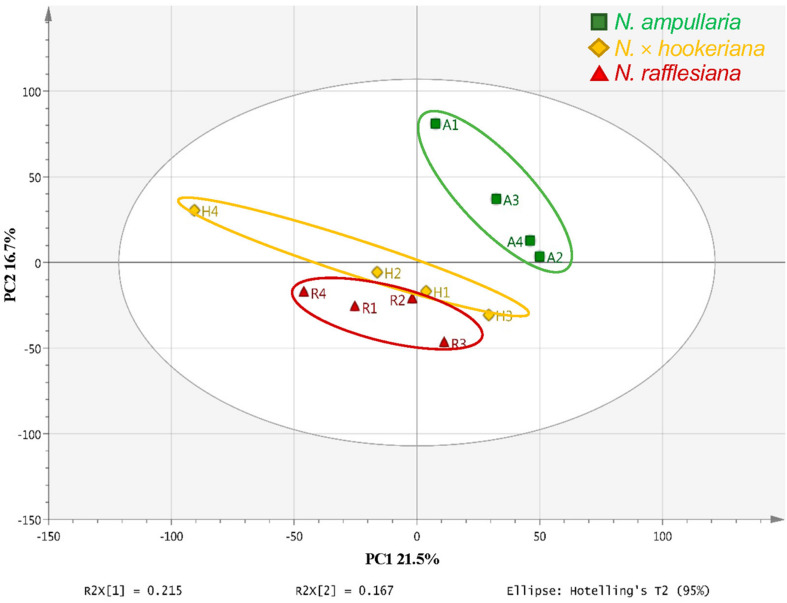
PCA score plot of pitcher extracts from different *Nepenthes* species.

An unsupervised principal component analysis (PCA) analysis shows the projections of each sample to other samples in a multidimensional space with each point representing an individual sample. The sample dispersions are related to the differences in metabolite compositions such that samples with higher similarities are closer together while samples with bigger differences are further apart. The parent clusters of *N. ampullaria* and *N. rafflesiana* were separated from each other, but the hybrid cluster was dispersed and closer to *N. rafflesiana* (Figure 4). This separation only accounted for 21.5% of variation on the first principal component, and the cumulative values of R^2^ and Q^2^ were lower than 0.5 despite having no outlier. This indicates high variations between biological replicates, which could be due to environmental factors since sampling was performed on-site in the field.

**FIGURE 5:**
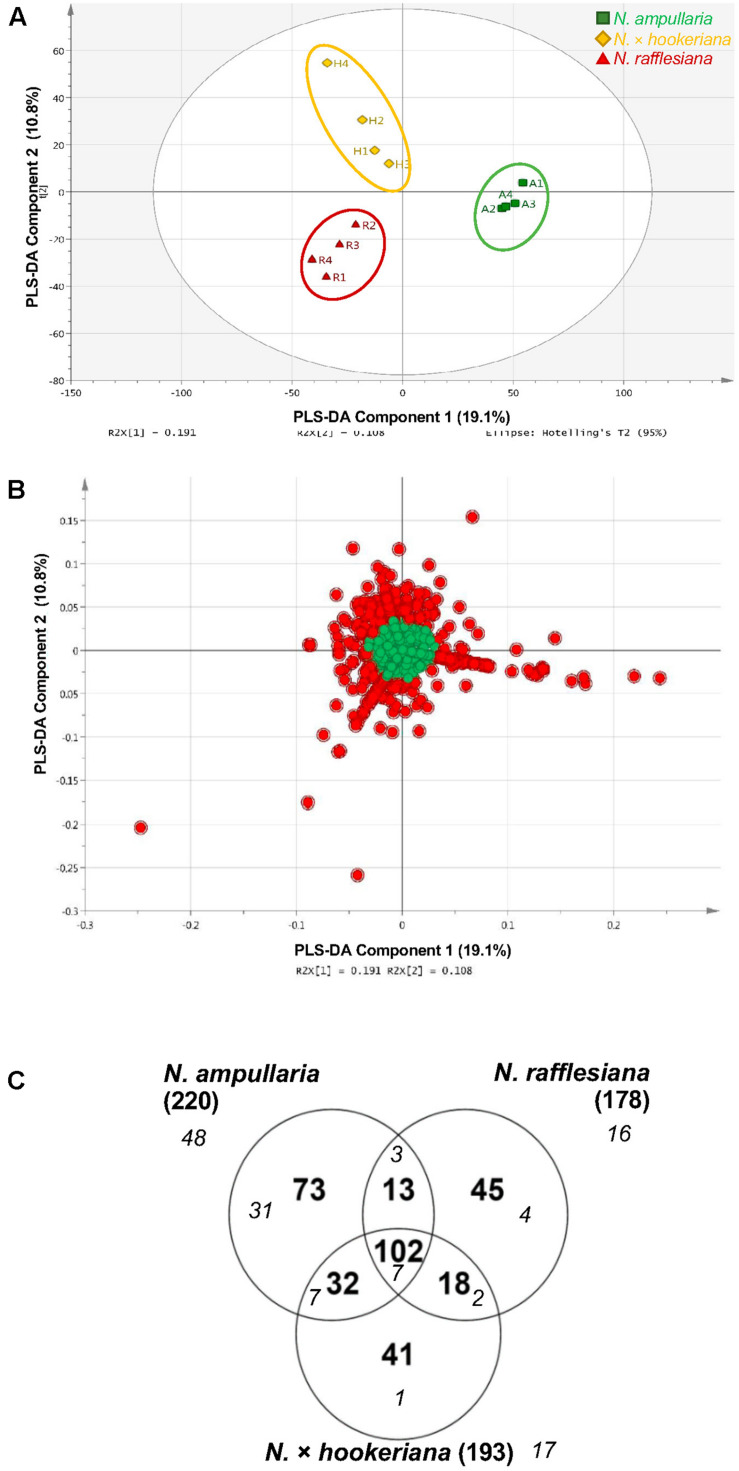
PLS-DA analysis of pitcher extracts from different *Nepenthes* species. **(A)** PLS-DA score plot. **(B)** PLS-DA loading plot with red dots showing the VIP features. **(C)** Venn analysis of VIP features in three *Nepenthes* species. Numbers in parentheses show the total number for each species. Italic numbers in smaller font size represent the numbers of significant (*P* < 0.05) VIP.

A supervised partial least squares-discriminant analysis (PLS-DA) was then performed for sample clustering with species information (Figure 5A). *N. ampullaria* samples were closely clustered and separated from *N. rafflesiana* along the first component that accounted for 19.1% variations. *N.* × *hookeriana* samples were more dispersed and closer to *N. rafflesiana* on the first component with separation mainly on the second component that explained 10.8% variations. These two components appeared to be a good fit model with a predictive value of confidence prediction (Q2) at 35% lower than the value of R2Y at 88%, which suggests no overfitting as supported by a permutation test (Supplementary Figure 5).

The metabolite features responsible for the discrimination between the three species can be observed in the PLS-DA loading plot (Figure 5B) with a higher contribution by the metabolites that are projected further from the center. A total of 324 (22.5%) metabolite features with variables important in projection (VIP) values of more than 1 were identified in the PLS-DA loading plot (Figure 5B), in which 102 VIPs were shared among the three species with *N. ampullaria* having the highest number of VIPs (Figure 5C). Apart from VIPs, univariate statistical analysis can identify statistically significant metabolites by comparing intensity differences of metabolite features in each sample, whereby only statistically significant features are considered important and maybe biological or chemical markers. Only 55 (17%) out of 324 VIPs were statistically significant (*P* < 0.05) based on the one-way variance analysis (ANOVA) in which 31 were unique to *N*. *ampullaria*. PCA performed based on these 55 significant VIPs can separate the three species into three distinct clusters and explained 63.2% of variation on the first principal component (PC1) and 14.2% for PC2 (Supplementary Figure 6). This supports that these metabolite features contribute significantly to the distinction of each species and account for their differences in metabolism.

**FIGURE 6:**
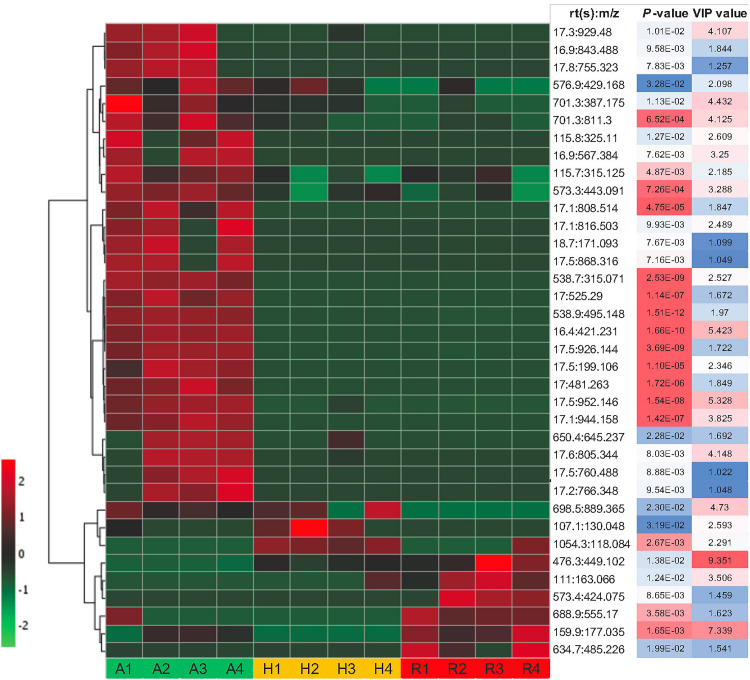
Heat map of statistically significant features in all three species. The color temperature represents the relative peak intensity with hotter red color showing higher intensity. Putative identification of metabolite features can refer to Supplementary File 1.

Some of these significant metabolite features were plotted in a heat map with normalized intensity values (Figure 6). A total of 27 out of the 36 metabolite features showed higher abundance in *N. ampullaria* compared to six metabolites in *N. rafflesiana* and only three in the hybrid. This indicates that *N. ampullaria* displayed a more distinct metabolite distribution compared to *N. rafflesiana* and *N.* × *hookeriana*, which is consistent with the results from the multivariate analysis.

### Metabolite Identification

Due to the labor-intensive metabolite identification, we mainly focused on VIP and metabolite features with high abundance for putative identification of metabolites by manually matching the accurate masses, fragmentation patterns, and MS/MS spectral data with reference to public metabolite databases, including METLIN, MassBank, and MetFrag (Supplementary File 1). For every annotated metabolite, we manually checked against the KNApSAcK database to make sure the metabolite is produced in plants. A total of 95 (29.3%) of 324 VIPs were among the 105 (7.3%) putatively identified metabolites from 1,441 metabolite features based on our manual approach.

## Discussion

### Untargeted Metabolite Profiling of *Nepenthes* Pitcher Extracts

This untargeted metabolite profiling study is the first comparative metabolomics analysis of *Nepenthes* pitchers from different species. For non-model organisms, LC-MS analysis is the preferred method for metabolic profiling with good selection and sensitivity to analyze various types of metabolites with large mass range differences and different physicochemical properties, such as volatility, heat loss, and polarity (Stobiecki and Kachlicki, 2013). The chromatographic separation can reduce the sample complexity and increase the MS detection sensitivity with less background noise. Furthermore, the electrospray ionization (ESI) mode of soft ionization can produce large amounts of ions through the exchange of charges in solution and often form intact molecular ions for initial identification of metabolites (Zhou et al., 2012). Time-of-flight (ToF) technology applied in this study further improves the mass accuracy to facilitate the identification and relative quantification of metabolites.

To compare the three *Nepenthes* species with four biological replicates of pitcher extracts from each species, their metabolite profile data were processed and normalized based on vanillin because it exhibited the most consistent peak intensities in all samples with the lowest coefficient of variation (CV) compared to ribitol and riboflavin (Supplementary File 1). Overall, multivariate analysis (MVA) showed that the metabolite composition of the hybrid is more like *N. rafflesiana* than *N. ampullaria*. This corroborates the recent report that the hybrid showed more similar transcriptome and secretome to *N. rafflesiana* than *N. ampullaria* (Zulkapli et al., 2021). Previously, it was proposed that the variation of colors and motifs were determined by male *Nepenthes* based on a genetic study (Yulita and Mansur, 2012). *N.* × *hookeriana* “spotted” as a hybrid between male *N. rafflesiana* and female *N. ampullaria* “green” was more like *N. rafflesiana* while *N.* × *hookeriana* “green” was hypothesized to be a hybrid between male *N. ampullaria* “green” and female *N. rafflesiana.* Hence, this explains the molecular compositions of samples in this study with *N.* × *hookeriana* “spotted” (Figure 1).

A recent untargeted metabolite profiling study of *N.* × *ventrata* pitchers was based on cheminformatics fingerprinting to infer metabolite classification without compound identification, which hindered functional interpretation (Dávila-Lara et al., 2020). At present, metabolite identification for untargeted metabolomics analysis largely depends on mass-based search using the m/z values of molecular ions of interest against databases (Xiao et al., 2012; Blaženović et al., 2018). In this study, manual searches against different public databases putatively identified a total of 105 metabolites with 95 VIPs in which only four metabolites were identified with MS/MS data. These putative metabolites are only suggestive because of limited information on *Nepenthes* metabolites in the public databases and the low hit of auto MS/MS spectra for most of the compounds. To our knowledge, this is the most comprehensive study of metabolite profiling ever reported in *Nepenthes* studies.

Due to the complexity and diversity of the chemical composition of metabolites, metabolite identification is still a big challenge in metabolomics analysis (Aretz and Meierhofer, 2016). Commonly, there are less than 20% identified compounds in untargeted analysis, such as the Metabolomics Workbench (Sud et al., 2016) or the European metabolomics repository MetaboLights (Haug et al., 2013) even for the model organisms. A comprehensive spectral database for LC-MS is almost impossible with various instruments, methods, and acquisition of metabolic data, such as collision, fragmentation, and various settings resulting in varied retention time (rt) and molecular fragments (Aretz and Meierhofer, 2016).

### Functional Implications of Putatively Identified Metabolites in *Nepenthes* Pitchers

The most significant VIP metabolite feature (476.3s:449.102) was the most abundant metabolite in the pitchers with its peak clearly detectable at the eighth minute with different intensities in each species showing the highest abundance in *N*. *rafflesiana*, intermediate in the hybrid, and the lowest in *N*. *ampullaria* (Figure 2). VIP refers to metabolites that contribute to the discrimination between samples, and metabolites with high VIP values can serve as biological or chemical markers (Beatriz et al., 2014). A MS/MS spectrum match search using the precursor m/z with MS/MS peaks (Supplementary Figure 7) found 476.3s:449.102 matches to kaempferol 3-glucoside (astragalin) and cyanidin 3-O-glucoside (chrysenthemin), both with a METLIN score of 100 and Δppm = 11. However, further fragment similarity search only found hit to astragalin at a fragment peak of m/z 287.054 with Δppm = 9 (Supplementary Figure 7D). Nonetheless, we cannot exclude the possibility that 476.3s:449.102 could be other isomers, kaempferol O-glucosides, or luteolin O-glucosides without further validation and structural elucidation using reference compounds and NMR. Astragalin is a flavonol involved in the biosynthesis of flavones and flavonols. Astragalin is a type of yellow pigment that could protect plants from UV radiation (Nakabayashi, 1955). Therefore, this compound may contribute to the pitcher color and confer protection from UV radiation (Lavola et al., 2003). Astragalin is associated with various pharmacological and biological activities, including anti-inflammatory, antioxidant activity, anti-atopic dermatitis activity, TNF-a, IL-1b, and IL-6 production inhibiting activity, and inhibiting histamine release in human blood cells (Riaz et al., 2018).

This finding agrees with Kováčik et al. (2012) that found phenols constituted the most abundant metabolites in *Nepenthes*. The results of several other phytochemical studies also emphasized phenolic compounds in *Nepenthes* roots and leaves, particularly naphthoquinones (Cannon et al., 1980; Aung et al., 2002; Van Thanh et al., 2015). Flavonoids were found to be the dominant phenols in the pitcher extracts, apart from quinones, quinic acids, monolignols, lignans, and phenylpropanoids (Supplementary File 1). These flavonoids have antioxidant properties that protect plants from environmental stresses, especially oxidative stress and ultraviolet (UV) light (Pollastri and Tattini, 2011). Flavonoids involved in UV light protection include flavones and flavonols that also act as antioxidants in protecting plants from oxidative stress caused by temperatures higher than suboptimal temperatures (Herrmann, 1976; Jaakola and Hohtola, 2010). Flavan is a product of a double reduction of flavanone. Most of the flavans are soluble in lipids, making these compounds often found on the fruit peel and the cutin layer of the leaf surface (Swanson, 2003). According to Mierziak et al. (2014), the strongest antifungal activity is shown by unmodified flavones and flavanones. Therefore, these compounds are commonly used in plants as phytoalexins to fight fungi and insects (Swanson, 2003). Chalcones are usually intermediates in flavonoid biosynthesis and generally have low compositions in most plants but display a variety of biological activities as traditional medicine (Rozmer and Perjési, 2016).

Anthocyanins are responsible for color in plants. Cyanidin and delphinidin are parts of anthocyanins that contribute to a variety of colors, including orange, red, and purple, depending on the types, levels, and structural modifications of anthocyanins, the presence of co-pigments such as flavones or flavonol, and pH variations (Yu and McGonigle, 2005). Anthocyanidins were present in pitchers of all species but in relatively low abundance (Supplementary File 1). These compounds may be involved in the pitcher pigmentation and coloration, such as spots on the pitchers.

The presence of flavonols and flavones, which were highly abundant in *N. rafflesiana*, may also contribute to the red coloration of pitchers, although the distribution of anthocyanidins in the species is low (Supplementary File 1). A high temperature can inhibit anthocyanin biosynthesis caused by chemical degradation from the accumulation of polyphenol oxidase and peroxide enzymes (Vaknin et al., 2005; Mori et al., 2007). This partly explains the effect of latitude on plant flavonoid biosynthesis (Jaakola and Hohtola, 2010). For example, a tropical herb plant *Polygonum minus* growing in the lowland and exposed to higher temperatures contained higher amounts of flavones and flavonols but lower anthocyanidin content compared to those from highland and exposed to lower temperatures (Goh et al., 2016). However, whether or not *N. rafflesiana* plants growing at lowland and highland exhibit different compositions of anthocyanidins remains to be investigated.

In previous reports, phenolic compound naphthoquinones such as plumbagin with numerous bioactivities are known to be highly abundant in *Nepenthes* (Likhitwitayawuid et al., 1998; Gwee et al., 2014). Plumbagin has been suggested with a possible role in attracting their insect prey with distinct volatile preferences toward the sticky trap of another insectivorous plant, *Drosera auriculata* (El-Sayed et al., 2016; Widhalm and Rhodes, 2016). According to Shin et al. (2007), some naphthoquinones with antifungal activities can be found in the roots of *N. rafflesiana*, *N. thorelii*, *N. gracilis*, and *N. insignis*. Studies have also shown that naphthoquinones can be found in the leaves of *N. khasiana* and *N. gracilis* (Aung et al., 2002; Eilenberg et al., 2010; Raj et al., 2011). Naphthoquinones might have several functions in *Nepenthes*, including photosynthesis, pigmentation and coloration, and protection against UV light, desiccation, and insects (Widhalm and Rhodes, 2016). Raj et al. (2011) speculated toxic or anesthetic effects of naphthoquinones on insect prey in *N. khasiana* pitchers. Furthermore, derivatives of naphthoquinones, droserone, and 5-O-methyl droserone could protect and maintain the sterility of the pitcher fluids against microbes from the trapped prey and together with plumbagin may act as molecular triggers in prey capture and digestion (Raj et al., 2011).

In this study, there were a few naphthoquinones putatively identified (Supplementary File 1), which have never been reported. There might be several factors that contribute to the detection of naphthoquinones, including the differences in the extraction using different solvents or analytical methods such as GC-MS (Raj et al., 2011). Besides that, Eilenberg et al. (2010) showed that naphthoquinones in *N. khasiana* pitcher fluids only present after chitin induction, which suggests the induced production of naphthoquinones.

As mentioned above, this study is limited by the uncertain metabolite identification for a detailed discussion of metabolites in *Nepenthes* pitchers. Furthermore, this also hindered further discussion on whether the different metabolite compositions in the pitchers were accountable by the different dietary habits of the parent and hybrid species. Nevertheless, this study is meaningful in finding potential chemical markers and bioactive compounds. Various reports described the curative effects of extracts from *Nepenthes* on diseases, for example, jaundice, hepatitis, gastric ulcers, ureteral stones, diarrhea, diabetes, cough, fever, hypertension, urinary system infections (Thao et al., 2016), malaria (Likhitwitayawuid et al., 1998), and Staphylococcus infection (Wiart et al., 2004), and recently on different kinds of oral cancer cells (Tang et al., 2019). Thus, further works on the validation of metabolites from *Nepenthes* as well as the analysis of their putative pharmaceutical uses are promising to explore new compounds and therapeutics. Subsequently, compounds with bioactivities of interest can be targeted for isolation, structural elucidation, and functional characterization.

## Conclusion

This study provides a comprehensive profiling and putative identification of metabolites from *Nepenthes* pitchers through the untargeted LC-MS approach with a total of 1,441 metabolite features and 105 putatively identified metabolites. MVA performed on profiled metabolites showed that the chemical compositions of hybrid pitchers were more similar to that of *N. rafflesiana* than *N. ampullaria*. Astragalin, which is a pharmacologically useful flavonol, was putatively identified as the most significant compound in distinguishing between the three *Nepenthes* species with potential as a chemical marker. However, the influence of dietary habit on the differences in the metabolite compositions between the three taxa remains unclear.

## Data Availability Statement

The original contributions presented in the study are included in the article/Supplementary Material, further inquiries can be directed to the corresponding author/s.

## Author Contributions

MR and H-HG conceived the study and experiments. MR performed the experiments and analyzed data. MR, AM, KA, SB, and H-HG discussed the data and wrote the manuscript. H-HG acquired the funding for this work. All authors read and agreed to the present version of the manuscript.

## Conflict of Interest

The authors declare that the research was conducted in the absence of any commercial or financial relationships that could be construed as a potential conflict of interest.

## Footnotes

1 https://metlin.scripps.edu/

2 http://www.massbank.jp/Search

3 https://msbi.ipb-halle.de/MetFrag/

4 https://xcmsonline.scripps.edu/

5 http://www.metaboanalyst.ca

6 http://bioinformatics.psb.ugent.be/webtools/Venn/

## Supplementary Material

Supplementary Figure 1 *Nepenthes* pitcher plants in this study. **(A–C)** *Nepenthes ampullaria.* **(D,E)** *Nepenthes* × *hookeriana.* **(F–H,J,K)** *Nepenthes rafflesiana* **(A,D,F)** Whole pitcher plants. **(B)** A cluster of *N. ampullaria* pitchers on the ground. **(C,E,H)** A single pitcher. **(G)** An aerial pitcher of *N. rafflesiana*. **(I)** Experimental terrace for pitcher sampling of all three species. **(J)** Characteristics of a typical *Nepenthes* pitcher. **(K)** External and internal structures of a *N. rafflesiana* pitcher.

Supplementary Figure 2 **(A)** Measurement of pitcher length. **(B)** Pitcher length at each growth phase. Error bars showing standard errors of six biological replicates.

Supplementary Figure 3 Mirror cloud plots of differentially accumulated features (DAFs) in pairwise comparisons of **(A)** *N. ampullaria* and *N. rafflesiana*, **(B)** *N. ampullaria* and *N.* × *hookeriana*, and **(C)** *N. rafflesiana* and *N.* × *hookeriana*. The numbers of significant (*P* < 0.001, rt > 1) DAFs are shown in parentheses and listed on the side with details in ascending *P*-value. DAFs are shown for lower (top) or higher (bottom) accumulation in the first species of comparison. Circle size depicts the average fold change. Y-axis indicates the m/z value of the feature; the further away from the origin, the higher the m/z. The superimposed, raw base-peak chromatograms (BPC) of all runs are shown in the background, colored according to different samples. Insets show peak intensity boxplots of selected DAFs with m/z values.

Supplementary Figure 4 Venn analysis of identified features in XCMS pairwise comparisons of all three species. A: *N. ampullaria*; H: *N.* × *hookeriana*; R: *N. rafflesiana.*

Supplementary Figure 5 Permutation test of PLS-DA validation model.

Supplementary Figure 6 PCA score plot analysis based on significant features. Each symbol represents different species of *Nepenthes*, green square: *N. ampullaria*, yellow diamond: *N.* × *hookeriana*, and red triangle: *N. rafflesiana.*

Supplementary Figure 7 Putative identification of metabolite feature 476.3s:449.102 based on tandem MS data. **(A)** MS/MS spectra of 476.3s:449.102 with the list of peaks (m/z and intensity). **(B)** Chemical structure of astragalin and chrysenthemin. Comparison for putative metabolite identification based on **(C)** METLIN MS/MS spectrum and **(D)** Fragment Similarity Search. The blue square in **(A)** shows the precursor ion. For **(C)**, the asterisk indicates the highest peak of precursor ion, while the black and cyan lines represent the MS/MS spectrum of the putative metabolite and the search fragments, respectively.

Supplementary File 1 Normalized peak intensity values of metabolite features with putative metabolite identification.
