# Supplementary material for: UPLC-TOF-MS/MS-Based Metabolomics Analysis Reveals Species-Specific Metabolite Compositions in Pitchers of Nepenthes ampullaria, Nepenthes rafflesiana, and Their Hybrid Nepenthes × hookeriana
Source: Front Plant Sci. 2021 Apr 21;12:655004. doi: 10.3389/fpls.2021.655004 (PMC8096924; doi:10.3389/fpls.2021.655004)
Supplement: Supplementary Figure 1 — Nepenthes pitcher plants in this study. (A–C) Nepenthes ampullaria. (D,E) Nepenthes × hookeriana. (F–H,J,K) Nepenthes rafflesiana (A,D,F) Whole pitcher plants. (B) A cluster of N. ampullaria pitchers on the ground. (C,E,H) A single pitcher. (G) An aerial pitcher of N. rafflesiana. (I) Experimental terrace for pitcher sampling of all three species. (J) Characteristics of a typical Nepenthes pitcher. (K) External and internal structures of a N. rafflesiana pitcher. [file Data_Sheet_2.pdf]

UPLC-TOF-MS/MS-based untargeted metabolomics analysis reveals species-specific metabolite compositions in pitchers of *Nepenthes ampullaria*, *Nepenthes rafflesiana*, and their hybrid *Nepenthes* × *hookeriana*

*Muhammad Aqil Fitri Rosli, Ahmed Mediani, Kamalrul Azlan Azizan, Syarul Nataqain Baharum and Hoe-Han Goh**

E-mail: gohhh@ukm.edu.my

Supplementary Material

Supplementary Figure 1 I *Nepenthes* pitcher plants in this study. (**A-C**) *Nepenthes ampullaria.* (**D-E**) *Nepenthes* x *hookeriana.* (**F-H, J, K**) *Nepenthes rafflesiana* (**A, D, F**) Whole pitcher plants. (**B**) A cluster of *N. ampullaria* pitchers on the ground. (**C, E, H**) A single pitcher. (**G**) An aerial pitcher of *N. rafflesiana*. (**I**) Experimental terrace for pitcher sampling of all three species. (**J**) Characteristics of a typical *Nepenthes* pitcher. (**K**) External and internal structures of a *N. rafflesiana* pitcher.

Supplementary Figure 2 I (**A**) Measurement of pitcher length. (**B**) Pitcher length at each growth phase. Error bars showing standard errors of six biological replicates.

Supplementary Figure 3 I Mirror cloud plots of differentially accumulated features (DAFs) in pairwise comparisons of (**A**) *N. ampullaria* and *N. rafflesiana,* (**B**) *N. ampullaria* and *N.* × *hookeriana*, and (**C**) *N. rafflesiana* and *N.* × *hookeriana*. The numbers of significant (P<0.001, rt > 1) DAFs are shown in parentheses and listed on the side with details in ascending P-value. DAFs are shown for lower (top) or higher (bottom) accumulation in the first species of comparison. Circle size depicts the average fold change. Y-axis indicates the m/z value of the feature; the further away from the origin, the higher the m/z. The superimposed, raw base-peak chromatograms (BPC) of all runs are shown in the background, colored according to different samples. Insets show peak intensity boxplots of selected DAFs with m/z values.

Supplementary Figure 4 I Venn analysis of identified features in XCMS pairwise comparisons of all three species. A: *N. ampullaria*; H: *N.* × *hookeriana*; R: *N. rafflesiana.*

Supplementary Figure 5 I Permutation test of PLS-DA validation model.

Supplementary Figure 6 I PCA score plot analysis based on significant features. Each symbol represents different species of *Nepenthes*, green square: *N. ampullaria*, yellow diamond: *N.* x *hookeriana*, and red triangle: *N. rafflesiana.*

Supplementary Figure 7 I Putative identification of metabolite feature 476.3s:449.102 based on tandem MS data. (**A**) MS/MS spectra of 476.3s:449.102 with the list of peaks (m/z and intensity). (**B**) Chemical structure of astragalin and chrysenthemin. Comparison for putative metabolite identification based on (**C**) METLIN MS/MS spectrum and (**D**) Fragment Similarity Search. The blue square in (**A**) shows the precursor ion. For (**C**), the asterisk indicates the highest peak of precursor ion, while the black and cyan lines represent the MS/MS spectrum of the putative metabolite and the search fragments, respectively.

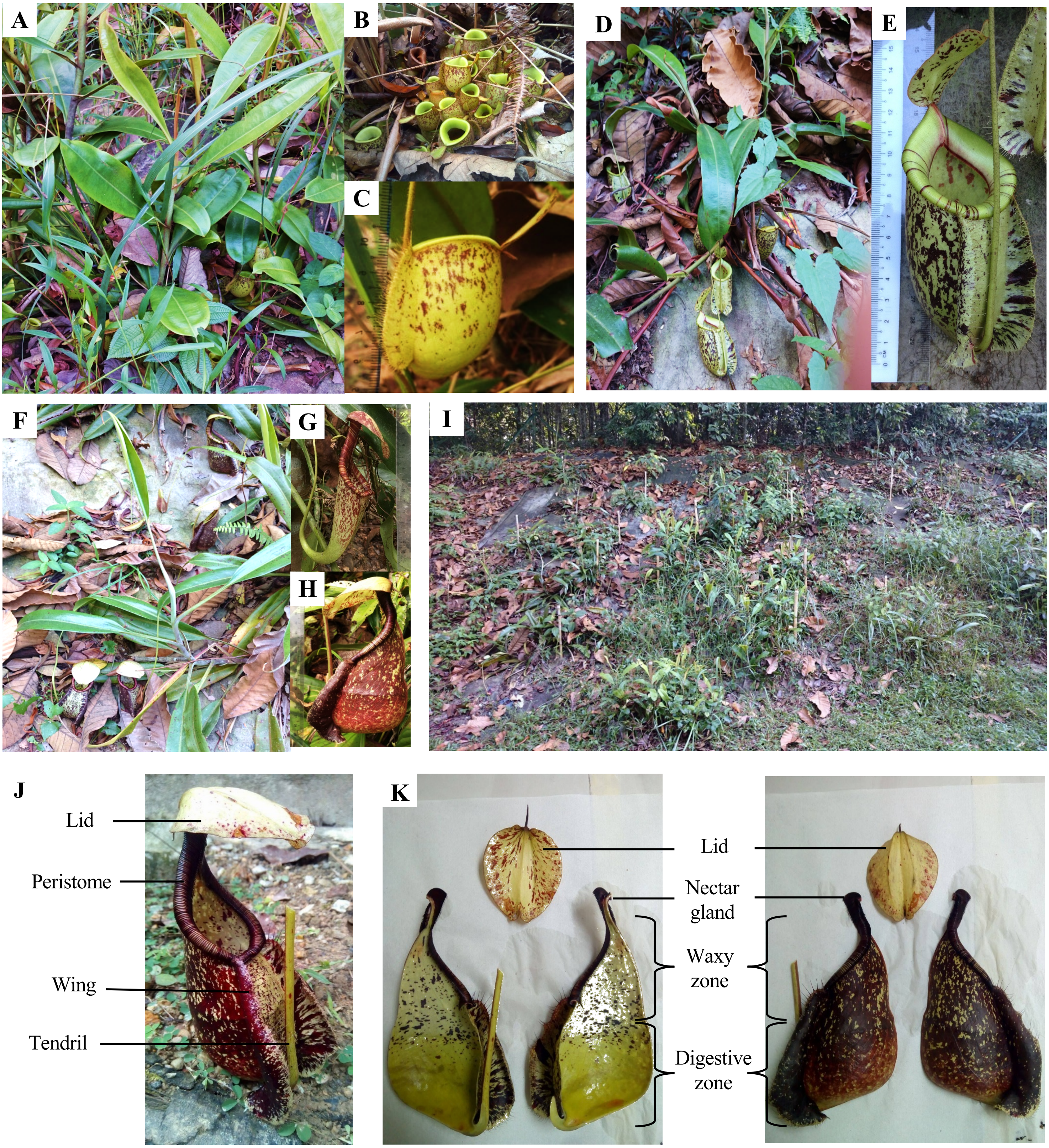

Supplementary Figure 1 l *Nepenthes* pitcher plants in this study. (**A-C**) *Nepenthes ampullaria.* (**D-E**) *Nepenthes* x *hookeriana.* (**F-H, J, K**) *Nepenthes rafflesiana* (**A, D, F**) Whole pitcher plants. (**B**) A cluster of *N. ampullaria* pitchers on the ground. (**C, E, H**) A single pitcher. (**G**) An aerial pitcher of *N. rafflesiana.* (**I**) Experimental terrace for pitcher sampling of all three species. (**J**) Characteristics of a typical *Nepenthes* pitcher. (**K**) External and internal structures of a *N. rafflesiana* pitcher.

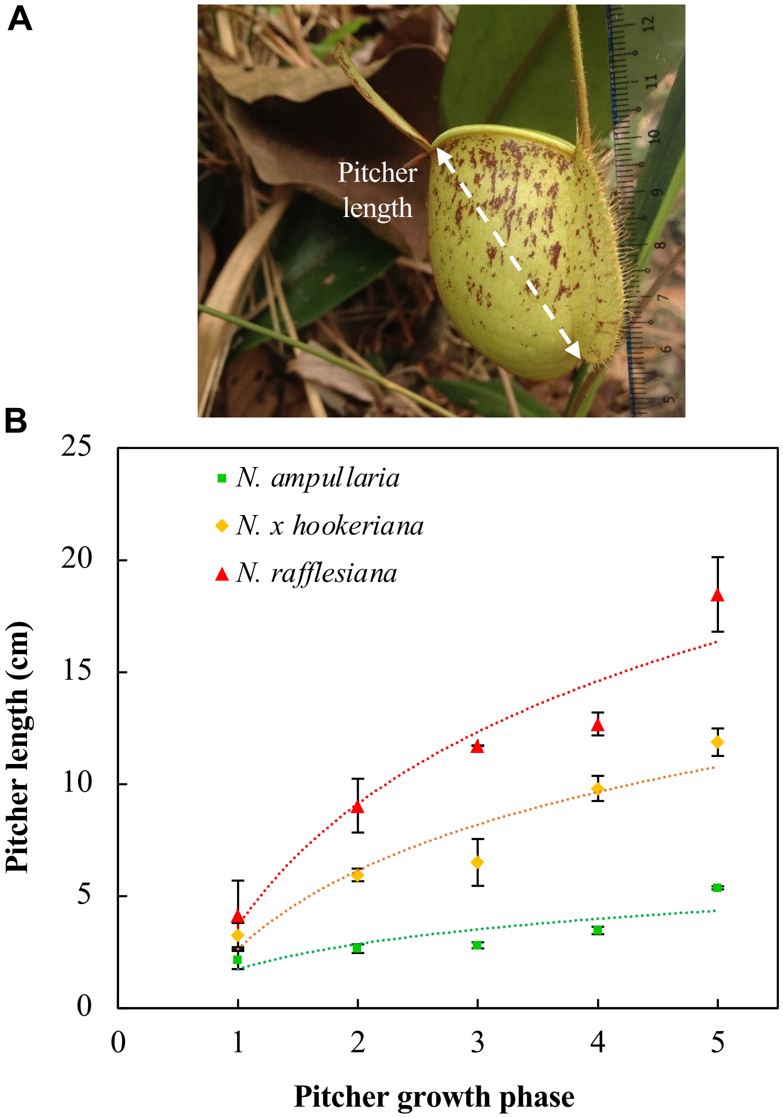

Supplementary Figure 2 I (**A**) Measurement of pitcher length. (**B**) Pitcher length at each growth phase. Error bars showing standard errors of six biological replicates.

A

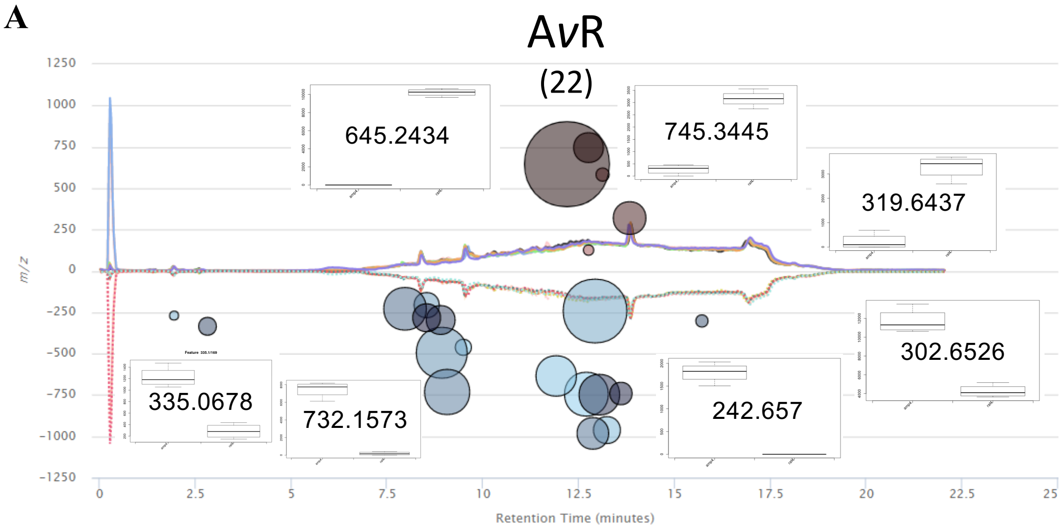

fold	pvalue	updown	mzmed	rtmed
1067.2	0.00002	UP	645.2434	12.2
11.5	0.00003	UP	745.3445	12.76
14.6	0.0001	UP	319.6437	13.85
3	0.00031	UP	582.2417	13.13
2.3	0.00041	UP	125.5702	12.77
9.8	0.00004	DOWN	283.2592	8.53
6.3	0.00009	DOWN	741.4681	13.62
28.1	0.00014	DOWN	748.3556	13.06
10.7	0.00022	DOWN	297.0663	8.92
4.3	0.00024	DOWN	335.0678	2.82
2.7	0.00029	DOWN	302.6526	15.73
33.1	0.00031	DOWN	229.2085	7.98
13.5	0.00035	DOWN	982.4596	12.89
40.7	0.00037	DOWN	732.1573	9.07
65.4	0.00043	DOWN	495.1462	8.94
7.9	0.00043	DOWN	207.1331	8.53
187.7	0.00049	DOWN	242.657	12.94
2.1	0.0007	DOWN	270.0629	1.94
9.1	0.00071	DOWN	962.4543	13.24
27.7	0.00083	DOWN	635.2543	11.91
36.5	0.00088	DOWN	745.3618	12.72
3.8	0.00097	DOWN	462.0656	9.51

B

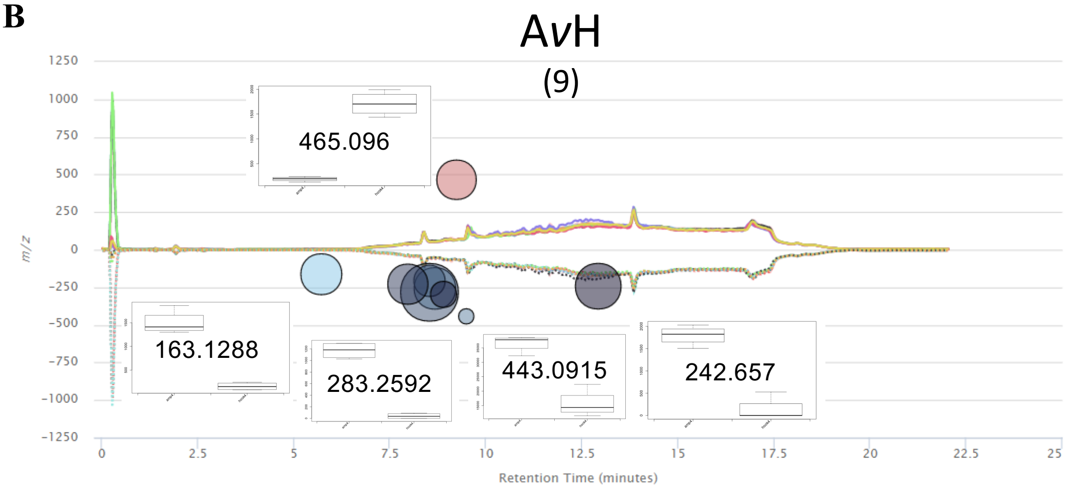

fold	pvalue	updown	mzmed	rtmed
12.8	0.00008	DOWN	242.657	12.94
4.3	0.0001	DOWN	297.0663	8.92
9.4	0.00014	DOWN	229.2085	7.98
24.9	0.00018	DOWN	283.2592	8.53
5.7	0.0003	DOWN	207.1331	8.53
2.3	0.0006	DOWN	443.0915	9.51
10.1	0.00079	DOWN	257.2396	8.69
9	0.00081	UP	465.096	9.24
9.8	0.00089	DOWN	163.1288	5.73

C

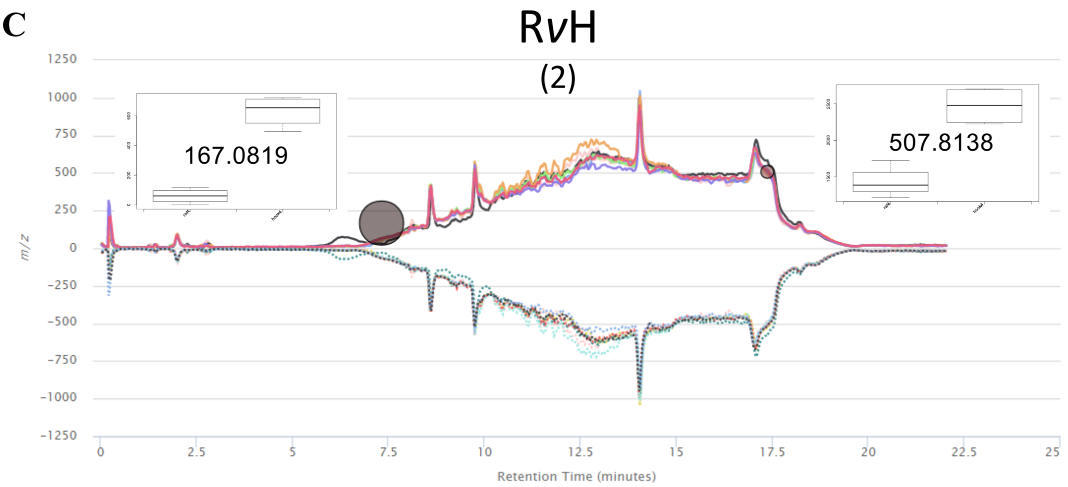

fold	pvalue	updown	mzmed	rtmed
6.5	0.00041	UP	167.0819	7.32
1.7	0.00095	UP	507.8138	17.39

Supplementary Figure 3 I Mirror cloud plots of differentially accumulated features (DAFs) in pairwise comparisons of (**A**) *N. ampullaria* and *N. rafflesiana,* (**B**) *N. ampullaria* and *N.* × *hookeriana*, and (**C**) *N. rafflesiana* and *N.* × *hookeriana*. The numbers of significant (*P*<0.001, rt > 1) DAFs are shown in parentheses and listed on the side with details in ascending *P*-value. DAFs are shown for lower (top) or higher (bottom) accumulation in the first species of comparison. Circle size depicts the average fold change. Y-axis indicates the m/z value of the feature; the further away from the origin, the higher the m/z. The superimposed, raw base-peak chromatograms (BPC) of all runs are shown in the background, colored according to different samples. Insets show peak intensity boxplots of selected DAFs with m/z values.

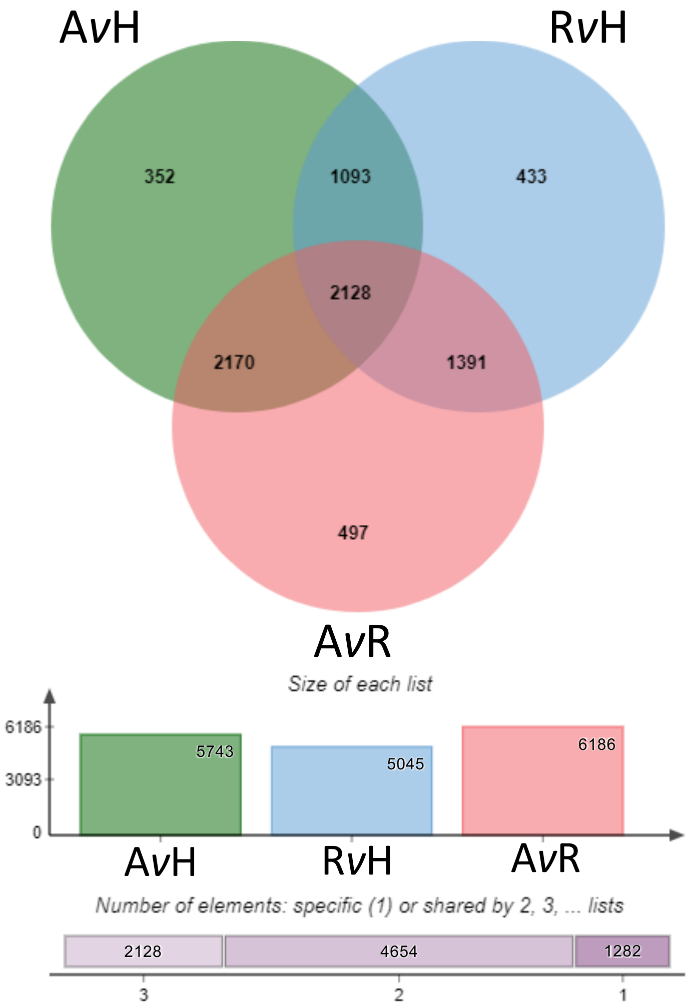

Supplementary Figure 4 I Venn analysis of identified features in XCMS pairwise comparisons of all three species. A: *N. ampullaria*; H: *N. × hookeriana*; R: *N. rafflesiana.*

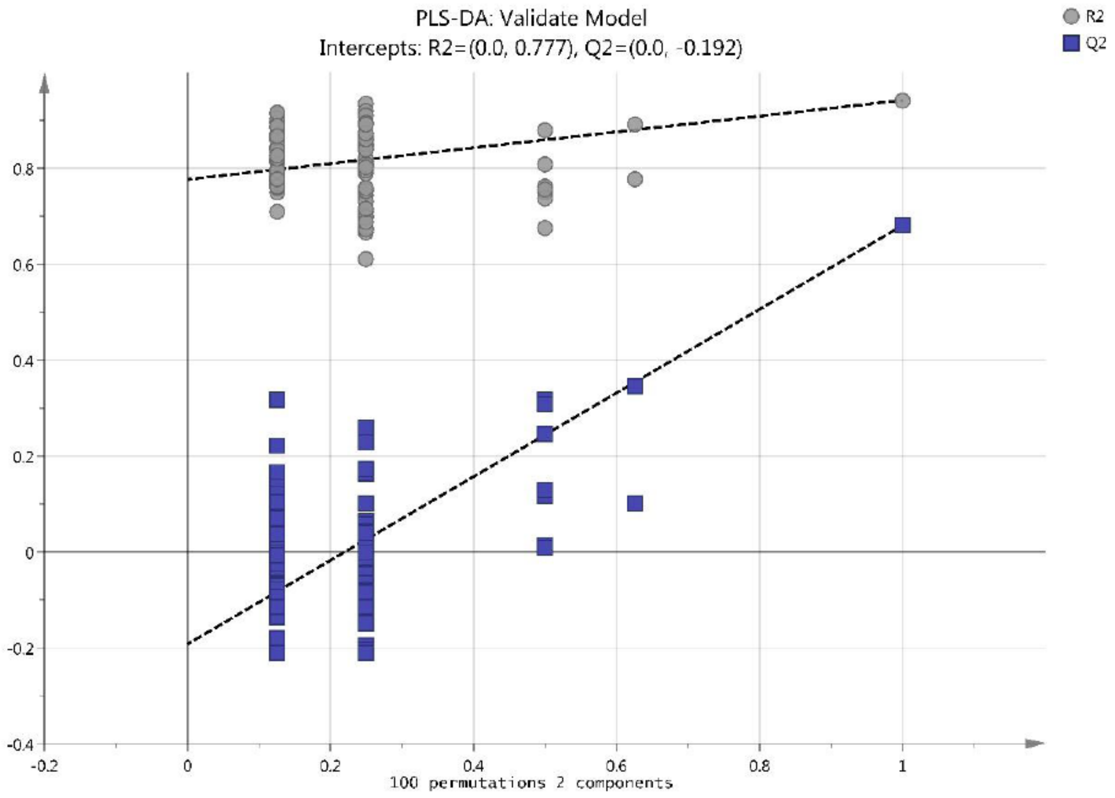

Supplementary Figure 5 l Permutation test of PLS-DA validation model.

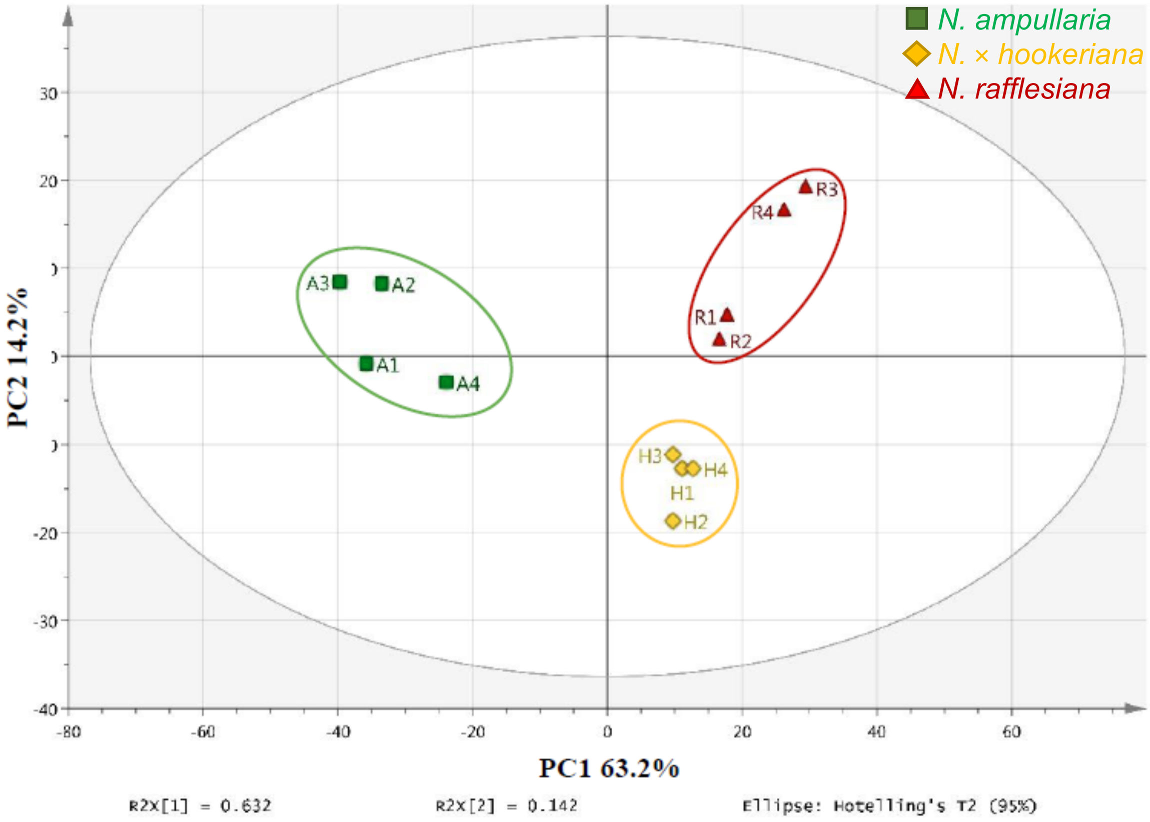

Supplementary Figure 6 l PCA score plot analysis based on significant features. Each symbol represents different species of *Nepenthes*, green square: *N. ampullaria*, yellow diamond: *N.* x *hookeriana*, and red triangle: *N. rafflesiana.*

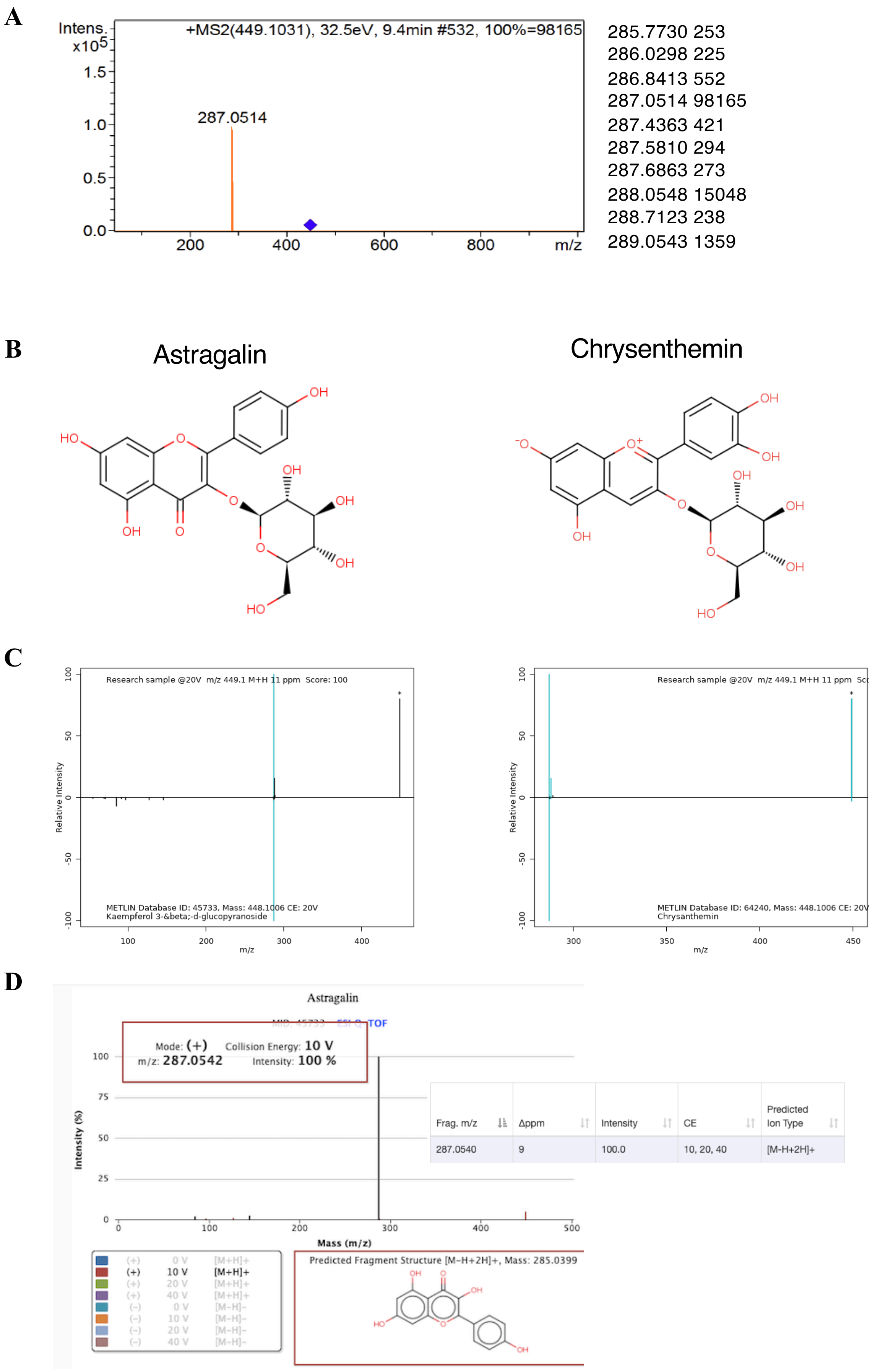

Supplementary Figure 7 I Putative identification of metabolite feature 476.3s:449.102 based on tandem MS data. (**A**) MS/MS spectra of 476.3s:449.102 with the list of peaks (m/z and intensity). (**B**) Chemical structure of astragalin and chrysenthemin. Comparison for putative metabolite identification based on (**C**) METLIN MS/MS spectrum and (**D**) Fragment Similarity Search. The blue square in (**A**) shows the precursor ion. For (**C**), the asterisk indicates the highest peak of precursor ion, while the black and cyan lines represent the MS/MS spectrum of the putative metabolite and the search fragments, respectively.
